# Predictive factors for the development of proteinuria in cancer patients treated with bevacizumab, ramucirumab, and aflibercept: a single-institution retrospective analysis

**DOI:** 10.1038/s41598-020-58994-5

**Published:** 2020-02-06

**Authors:** Yuko Kanbayashi, Takeshi Ishikawa, Yusuke Tabuchi, Koichi Sakaguchi, Yoshimi Ouchi, Eigo Otsuji, Koichi Takayama, Tetsuya Taguchi

**Affiliations:** 1 Department of Outpatient Oncology Unit, University Hospital, Kyoto Prefectural University of Medicine, Kyoto, Japan; 2 Department of Education and Research Center for Clinical Pharmacy, Osaka University of Pharmaceutical Sciences, 4-20-1 Nasahara, Takatsuki, 569-1094 Osaka Japan; 3 Division of Endocrine and Breast Surgery, Departments of Surgery, Kyoto Prefectural University of Medicine, Kyoto, Japan; 4 Department of Molecular Gastroenterology and Hepatology, Kyoto Prefectural University of Medicine, Kyoto, Japan; 5 Department of Pharmacy, University Hospital, Kyoto Prefectural University of Medicine, Kyoto, Japan; 6 Division of Digestive Surgery, Departments of Surgery, Kyoto Prefectural University of Medicine, Kyoto, Japan; 7 Department of Pulmonary Medicine, Kyoto Prefectural University of Medicine, Kyoto, Japan

**Keywords:** Risk factors, Chemotherapy

## Abstract

The development of proteinuria restricts the dose of anti-angiogenic agents, thereby reducing their efficacy. Thus, this retrospective study was undertaken to identify predictive factors of the development of angiogenesis inhibitor-induced proteinuria, and to elucidate if there is a difference in the likelihood of proteinuria among anti-angiogenic agents or cancer types, to help guide future strategies to improve the safety, efficacy, and quality of life of patients receiving chemotherapy. Between April 2014 and February 2019, 124 cancer patients at our outpatient chemotherapy center who were receiving chemotherapy with bevacizumab, ramucirumab, or aflibercept were enrolled. Variables related to the development of proteinuria were extracted from the patients’ clinical records and used for regression analysis. The level of the proteinuria was evaluated based on CTCAE version 5. Multivariate ordered logistic regression analysis was performed to identify predictive factors for the development of proteinuria. The Wilcoxon/Kruskal-Wallis test was used to identify significant differences between groups. Significant factors identified included systolic blood pressure (SBP) [odds ratio (OR) = 1.031, 95% confidence interval (CI) = 1.005–1.058; *P* = 0.0197], number of cycles (OR = 1.049, 95% CI = 1.018–1.082; *P* = 0.0019), and calcium channel blocker use (OR = 2.589, 95% CI = 1.090–6.146; *P* = 0.0311). There was no difference among the three anti-angiogenic agents (*P* = 0.4969) or among cancer types (*P* = 0.2726) in the likelihood of proteinuria. In conclusion, SBP, number of cycles, and calcium channel blocker use were identified as significant predictors of the development of angiogenesis inhibitor-induced proteinuria. There was no difference among the three anti-angiogenic agents or among cancer types.

## Introduction

Anti-angiogenic agents targeting the human vascular endothelial growth factor pathway, such as bevacizumab, ramucirumab, and aflibercept, significantly improve overall survival and progression-free survival in colorectal, lung, gastric, breast cancers and so on^1–5^. The toxicity profile of anti-angiogenic agents differs from those of conventional cytotoxic anticancer agents; use of anti-angiogenic agents is commonly associated with elevated blood pressure, proteinuria, and bleeding^1–5^. The development of proteinuria restricts the dose of anti-angiogenic agents, thereby reducing their efficacy^6,7^. However, the predictive factors for proteinuria have not been fully elucidated, and few studies have examined the differences in the development of proteinuria among anti-angiogenic agents or cancer types.

Thus, this retrospective study was undertaken to identify predictive factors of the development of angiogenesis inhibitor-induced proteinuria and to elucidate if there is a difference in the likelihood of proteinuria among anti-angiogenic agents or cancer types to help guide future strategies to improve the safety, efficacy, and quality of life (QoL) of patients receiving chemotherapy.

## Patients and Methods

### Study period and participants

Between April 2014 and February 2019, this study enrolled 139 colon cancer, gastric cancer, breast cancer, and lung cancer patients who were receiving chemotherapy with anti-angiogenic agents at our outpatient chemotherapy center. The Medical Ethics Review Committee of the Kyoto Prefectural University of Medicine approved this study (Approval No. ERB-C-867-2). All procedures were performed in accordance with the ethical standards of the Medical Ethics Review Committee of the Kyoto Prefectural University of Medicine and the 1964 Helsinki Declaration and its later amendments. No prospective studies with human participants or animals were performed by any of the authors for this article. Due to the retrospective nature of this work, informed consent was waived for the individual participants included in the study in accordance with the ethical standards of the Medical Ethics Review Committee of the Kyoto Prefectural University of Medicine.

### Extraction of variables

For the regression analysis of factors related to angiogenesis inhibitor-induced proteinuria, variables were extracted by manual abstraction from clinical records. Variables evaluated included factors that could potentially impact the development of proteinuria: demographic data (sex, age, height, weight, body mass index, and body surface area), Eastern Cooperative Oncology Group performance status, cancer type, presence of comorbidity (diabetes mellitus, hypertension), laboratory test values [albumin, serum creatinine, and estimated creatinine clearance, lactate dehydrogenase (LDH) and fibrinogen)], administration of anti-angiogenic agents (number of cycles, and doses of bevacizumab, ramucirumab, and aflibercept, respectively), medications that affect blood pressure [renin-angiotensin system (RAS) inhibitors, calcium channel blockers, and loop or thiazide diuretics], and vital signs [systolic blood pressure (SBP), diastolic blood pressure (DBP)]. The presence of hypertension was assessed by prescriptions for antihypertensive drugs. Creatinine clearance was estimated using the Cockcroft and Gault equation based on serum creatinine, sex, age, and weight. Clinical information was extracted before the first dose of bevacizumab, ramucirumab, or aflibercept. The level of angiogenesis inhibitor-induced proteinuria was evaluated based on the National Cancer Institute Common Terminology Criteria for Adverse Events (CTCAE) version 5.0.

### Statistical analysis

Explanatory variables were examined for multicollinearity (correlation coefficient |r| ≥ 0.7), since when correlations exist among the variables, this can lead to incorrect results of regression analyses.

Explanatory variables were selected based on correlation strength with the level of the angiogenesis inhibitor-induced proteinuria (objective variable) or clinical significance. First, univariate ordered logistic regression analysis between the outcomes and each potential explanatory variable was performed. Subsequently, a multivariate ordered logistic regression model was constructed by employing the stepwise selection procedure with the potential candidate variables. The model used a variable entry criterion of 0.25 and a variable retention criterion of 0.15. Ordered logistic regression analysis was employed, because the level of proteinuria was evaluated by a graded scale and multiple factors potentially involved as predictive factors for the development of proteinuria had to be evaluated simultaneously. Optimal cutoff thresholds were determined using receiver operating characteristic curve (ROC) analysis^8^. In many clinical trials, the equal or >2 proteinuria grade was the drug discontinuation criterion. Therefore, patients were divided in two groups depending the equal or >2 proteinuria grade or not. Furthermore, whether there was a difference in the development of proteinuria among these three anti-angiogenic agents or cancer types was analyzed. The Wilcoxon/Kruskal-Wallis test was used to identify significant differences between groups.

For all statistical analyses, values of *P* < 0.05 (2-tailed) were considered significant. All analyses were performed using JMP^®^ version 14.3.0. (SAS Institute, Cary, NC, USA).

## Results

Of the 139 patients who received chemotherapy with bevacizumab, ramucirumab, or aflibercept, 15 were excluded from this study: 3 were discontinued with only one course due to adverse events[1 patient had general fatigue, 2 patients had febrile neutropenia during chemotherapy (nab-paclitaxel plus ramucirumab and paclitaxel plus ramucirumab)] and 12 had insufficient data. Table 1 presents the clinical characteristics of the 124 enrolled patients, the potential variables related to the development of proteinuria, and the results of univariate analysis. Although fibrinogen was a significant predictor in univariate analysis, it was not used for multivariate analysis due to the large number of missing data (n = 84). Multicollinearity was observed in body height, body weight, body mass index (BMI) and body surface area (BSA). BSA, which was considered the most clinically relevant in cancer chemotherapy, was used for analysis. The forward stepwise selection procedure identified five variables (serum creatinine, SBP, number of cycles, calcium channel blockers, and RAS inhibitors).

**Table 1:** Patients’ characteristics, extracted variables, and results of univariate analyses (n = 124).

	Grade 0 (n = 72)	Grade 1 (n = 22)	Grade 2 (n = 25)	Grade 3 (n = 5)	*P* value	Odds ratio (95%CI)
**Demographic data**						
Male, n (%)	36 (50.0)	10 (45.5)	14 (56.0)	3 (60.0)	0.670	1.16 (0.58–2.31)
Age (y), median (range)	66.5 (28–87)	72 (41–82)	71 (52–85)	71 (37–81)	0.032*	1.04 (1.00–1.07)
Height (cm), median (range)	162.7 (142.8–178.6)	156.6 (140.6–183.5)	162.5 (144–173)	160.5 (149–171.3)	0.407	0.98 (0.95–1.02)
Weight (kg), median (range)	55.1 (31.1–94.1)	51.7 (37–103)	57.7 (42–94)	45 (30–66.3)	0.932	1.00 (0.97–1.03)
BMI (kg/m^2^), median (range)	21.0 (12.7–30.7)	21.8 (14.6–32.7)	22.2 (15.9–32.5)	17.8 (13.5–22.6)	0.606	1.02 (0.94–1.11)
BSA (m^2^), median (range)	1.58 (1.14–2.1)	1.50 (1.28–2.2)	1.58 (1.33–2.05)	1.43 (1.15–1.78)	0.699	0.71 (0.13–4.00)
PS (0/1/2)	31/37/4	11/10/1	13/12/0	3/1/1	0.311	0.73 (0.4–1.34)
**Cancer type**						
Lung, n (%)	11(15.3)	5(22.7)	1(4.0)	0	0.315	0.58 (0.20–1.69)
Colorectal, n (%)	34(47.2)	10(45.5)	16(64.0)	5(100.0)	0.060	1.96 (0.97–3.94)
Gastric, n (%)	14(19.4)	4(18.2)	3(12.0)	0	0.291	0.59 (0.22–1.57)
Breast, n (%)	13(18.1)	3(13.6)	5(20.0)	0	0.694	0.83 (0.33–2.11)
**Comorbidity**						
Hypertension, n (%)	21(29.2)	10(45.5)	13(52.0)	2(40.0)	0.037*	2.12 (1.05–4.30)
Diabetes mellitus, n (%)	12(16.7)	3(13.6)	9(36.0)	1(20.0)	0.126	1.91 (0.83–4.36)
**Laboratory test value**						
Serum creatinine, mg/dL, median (range)	0.69 (0.38–1.12)	0.67 (0.47–1.13)	0.73 (0.37–1.32)	0.50 (0.38–1.77)	0.015*	7.55 (1.49–38.3)
Creatinine clearance, mL/min, median (range)	78.4 (29.5–148.0)	70.5 (40.0–131.1)	69.0 (36.6–161.2)	80.4 (19.2–163.9)	0.152	0.99 (0.98–1.00)
Albumin, g/dL, median (range)	3.9 (2.3–4.7)	3.8 (2.7–4.5)	3.9 (3.1–4.6)	3.3 (2.9–4.1)	0.83	0.93 (0.46–1.85)
LDH, U/L, median (range)	229 (139–1528)	218 (158–558)	218 (146–366)	173 (162–194)	0.134	0.997 (0.994–1.001)
Fibrinogen, mg/dL, median (range), n = 84	347 (114–597) n = 51	370 (212–873) n = 16	390 (235–650) n = 13	446.5 (363–489) n = 4	0.019*	1.004 (1.001–1.008)
**Angiogenesis inhibitor**						
Bevacizumab, n (%)	44(61.1)	10(45.5)	18(72.0)	3(60.0)	0.796	1.10 (0.54–2.22)
Ramucirumab, n (%)	26(36.1)	10(45.5)	5(20.0)	2(40.0)	0.469	0.76 (0.37–1.59)
Aflibercept, n (%)	2(2.8)	2(9.1)	2(8.0)	0	0.365	2.01 (0.44–9.13)
Bevacizumab dose, mg/kg, mean ± SD	8.17 ± 2.98	8.75 ± 3.95	7.64 ± 2.77	8.33 ± 5.77	0.754	0.98 (0.85–1.13)
Ramucirumab dose, mg/kg, mean ± SD	8.5 ± 0	8.6 ± 0	8 ± 0.57	8 ± 0	0.341	0.69 (0.32–1.48)
Aflibercept dose, mg/kg, mean ± SD	4 ± 0	4 ± 0	3.6 ± 0.57	-	0.998	-
**Number of cycles**	8 (2–52)	9 (2–33)	17 (2–72)	13 (3–45)	0.003*	1.04 (1.02–1.07)
**Regimen**						
Platinum-containing, n (%)	3(4.2)	1(4.5)	0	1(20.0)	0.969	1.04 (0.18–5.87)
Taxane-containing, n (%)	32 (44.4)	10 (45.5)	7(28.0)	1(20.0)	0.155	0.59 (0.29–1.22)
Fluorouracil-containing, n (%)	34 (47.2)	10(45.5)	16 (64.0)	4 (80.0)	0.121	1.73 (0.87–3.47)
**Concomitant medication**						
RAS inhibitors, n (%)	9(12.5)	4(18.2)	7(28.0)	1(20.0)	0.010*	2.09 (0.87–5.04)
Calcium channel blockers, n (%)	11(15.3)	7(31.8)	11(44.0)	2(40.0)	0.003*	3.27 (1.5–7.1)
Loop or thiazide diuretics, n (%)	6(8.3)	1(4.5)	1(4.0)	1(20.0)	0.713	0.77 (0.2–3.04)
**Vital signs before administration**						
SBP, mmHg, median (range)	123 (89–151)	123 (98–156)	131 (100–177)	135 (124–152)	0.003*	1.04 (1.01–1.06)
DBP, mmHg, median (range)	73 (43–97)	73.5 (60–106)	73.5 (47–98)	79 (67–82)	0.238	1.02 (0.99–1.05)

CI, confidence interval; BMI, body mass index; BSA, body surface area; PS, Performance Status; RAS, renin-angiotensin system; SBP, systolic blood pressure; DBP, diastolic blood pressure.

*P < 0.05.

**Figure 1:**
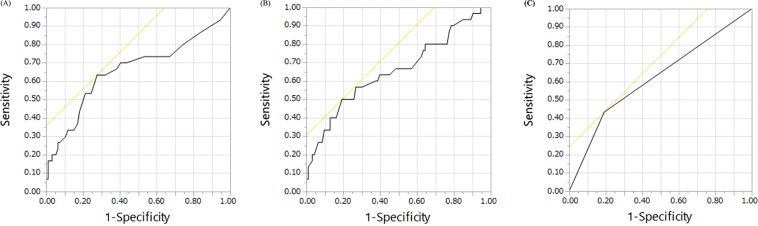
ROC curves about proteinuria (≥Grade 2) according the logistic regression significant variables. (**A**) ROC curve of the number of cycles with a sensitivity of 63.3% and specificity of 72.3% (AUC = 0.66). (**B**) ROC curve of systolic blood pressure with a sensitivity of 48.3% and specificity of 79.8% (AUC = 0.64). (**C**) ROC curve of calcium channel blockers with a sensitivity of 43.3% and specificity of 80.9% (AUC = 0.62).

These variables were then used for multivariate ordered logistic regression analysis. Significant factors identified for the development of proteinuria included SBP [odds ratio (OR) = 1.031, 95% confidence interval (CI) = 1.005–1.058; *P* = 0.0197], number of cycles (OR = 1.049, 95% CI = 1.018–1.082; *P* = 0.0019), and calcium channel blockers (OR = 2.589, 95% CI = 1.090–6.146; *P* = 0.0311) (Table 2). The accuracy of the model was 82/124, expressed as the ratio of patients whose expected value was equal to the observed value. The ROC curve analysis of the group likely to develop proteinuria (≥Grade 2) showed that the threshold for the number of cycles was ≥13, with a sensitivity of 63.3% and specificity of 72.3% [area under the curve (AUC) = 0.66](Fig. 1A), and that of SBP was ≥135 mmHg, with a sensitivity of 48.3% and specificity of 79.8% [AUC = 0.64](Fig. 1B). ROC curve of calcium channel blockers showed with a sensitivity of 43.3% and specificity of 80.9% (AUC = 0.62) (Fig. 1C).

**Table 2:** Results of multivariate ordered logistic regression analysis for variables extracted by forward selection (n = 124).

Variable	*P* value	Odds ratio	95%CI	
			Lower 95%	Upper 95%
Serum creatinine	0.1083	4.345	0.723	26.105
SBP, mmHg	0.0197*	1.031	1.005	1.058
Number of cycles	0.0019*	1.049	1.018	1.082
Calcium channel blockers	0.0311*	2.589	1.090	6.146
RAS inhibitors	0.8862	1.075	0.399	2.895

CI, confidence interval; SBP, systolic blood pressure; RAS, renin-angiotensin system.

*P < 0.05.

There was no difference in the development of proteinuria among the anti-angiogenic agents (bevacizumab, ramucirumab, and aflibercept; *P* = 0.4969), and there was also no difference among cancer types (colon, gastric, lung, and breast cancers; *P* = 0.2726).

## Discussion

The multivariate ordered logistic regression analysis performed in this study showed that the significant predictors for the development of proteinuria included number of cycles, SBP (before the initial administration of anti-angiogenic agents), and calcium channel blockers. Fibrinogen was also a predictor, as determined by univariate analysis. On ROC curve analysis of the potential factors responsible for the development of proteinuria, the cut-off value for the number of cycles was ≥13, and that of SBP was ≥135 mmHg. This study also showed that the likelihood of proteinuria was not different among anti-angiogenic agents or cancer types.

Several studies have reported that the development of angiogenesis inhibitor-induced proteinuria is dose-dependent^9–12^. The result of the current study is consistent with this previous finding. Thus, clinicians need to know that the incidence and severity of proteinuria increase as the number of administration cycles of anti-angiogenic agents increase, especially in patients with ≥13 cycles.

In the current study, the SBP cut-off value for the development of proteinuria was ≥135 mmHg. Previous studies demonstrated that high blood pressure is a major risk factor for proteinuria in the general population^13^. It has also been shown that SBP ≥130 mmHg is a risk factor for bevacizumab-induced proteinuria^14^; similarly, the present results showed that SBP ≥135 mmHg was a risk factor for proteinuria. Clinicians need to pay attention to blood pressure control before treatment.

Furthermore, the present study found that calcium channel blocker use is a risk factor for proteinuria. On the other hand, RAS inhibitor use was neither a protective factor nor a risk factor. As in previous research, the present results suggest that RAS inhibitor administration reduces the risk of proteinuria^15–18^. During treatment with anti-angiogenic agents, RAS inhibitors may be recommended for hypertension. Further research is needed on this issue.

Previous studies suggested that angiogenesis inhibitor-induced proteinuria is more likely to develop with colorectal cancer^15,19^. However, in the present study, there was no difference in the likelihood of proteinuria among cancer types. On the other hand, univariate analysis showed that proteinuria was more likely to occur in patients with colorectal cancer, but it was not significant on multivariate analysis. In patients with colorectal cancer, special attention may be needed regarding the development of proteinuria, but further study of this issue is needed. Fibrinogen was also a predictor, as determined by univariate analysis. This was consistent with previous studies^20^. Clinicians also need to pay attention elevated fibrinogen levels.

In the present study, there was no difference in the likelihood of proteinuria depending on anti-angiogenic agents. A previous study suggested that severe renal side effects may be less common with ramucirumab than with bevacizumab^21^. On the other hand, Peng *et al*. showed that the risk of developing all-grade and high-grade proteinuria was substantially higher with aflibercept than with bevacizumab^7^.

There were several limitations to the current study. First, the retrospective design of the research may have decreased the reliability of the data extracted. Second, since this study was conducted at a single institute, it only analyzed a relatively small number of patients. Therefore, prospective multicenter study will be needed to confirm these results.

In conclusion, SBP, number of cycles, and concomitant use of calcium channel blockers were identified as significant predictors of the development of proteinuria in cancer patients treated with bevacizumab, ramucirumab, or aflibercept. In addition, it was found that the likelihood of proteinuria was not different among anti-angiogenic agents or cancer types. However, these preliminary findings will need to be confirmed in a larger randomized controlled trial. There is a need to identify cancer patients who could develop side effects, in order to be able, in the future, to select them or/and treat them with more adequate doses of anti-angiogenic agents. Nevertheless, these findings may assist in developing chemotherapeutic strategies that can be used to improve the safety and efficacy including anti-angiogenic agents, as well as the QoL of patients.
